# Bacterial effector screening reveals RNF214 as a virus restriction factor in mammals

**DOI:** 10.1371/journal.ppat.1013035

**Published:** 2025-04-22

**Authors:** Aaron Embry, David F. Schad, Emily A. Rex, Neal M. Alto, Don B. Gammon

**Affiliations:** Department of Microbiology, University of Texas Southwestern Medical Center, Dallas, Texas, United States of America; Rutgers New Jersey Medical School, United States of America

## Abstract

Arboviruses are arthropod-borne viruses that pose significant threats to human and animal health. Previously, we demonstrated that bacterial effector proteins can serve as molecular tools to identify host immunity factors in insect cells that restrict arbovirus replication. In this study, we apply our bacterial effector screening system to identify immunity factors in two mammalian hosts—bats and humans. Our screens identified three bacterial effectors (IpaH4, SopB and SidM) that enhanced the replication of unrelated arboviruses in bat and human cells. We also discovered several effectors that enhanced arbovirus replication in an arbovirus- or host-specific manner. Focusing on the *Shigella flexneri*-encoded E3 ubiquitin ligase IpaH4, we identified the uncharacterized mammalian really interesting new gene (RING)-domain-containing protein RNF214 as a direct target that is ubiquitinated and degraded by IpaH4. RNF214 belongs to a large family of RING finger (RNF) proteins that primarily function as E3 ubiquitin ligases and that have diverse roles in regulating and mediating innate immune responses to disparate pathogens. Phylogenetic analyses reveal that RNF214 is highly conserved across vertebrate species, suggesting a conserved role in host defense. Functional studies demonstrate that RNF214 overexpression suppresses arbovirus infection in a manner dependent on its putative E3 ubiquitin ligase activity, whereas RNF214 depletion enhances viral replication in both human and bat cells. Furthermore, knockout of RNF214 did not alter the upregulation of interferon (IFN)-stimulated gene expression during infection or upon treatment of cells with IFN. Screening of 11 RNA and DNA viruses, revealed that RNF214 specifically restricts single-stranded RNA (ssRNA) viruses. These findings establish RNF214 as a critical component of the innate immune response against ssRNA viruses that may function independently of the IFN response. More broadly, our work highlights the utility of bacterial effector proteins as powerful tools for uncovering novel antiviral machinery in mammals.

## Author Summary

Arboviruses are transmitted by biting insects to animal and human hosts and cause diseases ranging from mild febrile illnesses to fatal encephalitis. Here, we use bacterial effector proteins to identify mammalian factors that block arbovirus replication. Bacterial effectors encoded by pathogenic bacteria are secreted into mammalian host cells to inhibit cellular antimicrobial responses. Like viruses, many pathogenic bacteria replicate inside of mammalian host cells, thus we hypothesized that some bacterial effectors may target and inhibit host immune responses that are restrictive to both bacteria and viruses. After screening a library of >200 bacterial effectors, we identified three effectors that promote the replication of arboviruses belonging to two distinct viral families in bat and human cells. We further show that one of our most potent effectors, IpaH4, enhances arbovirus replication by targeting mammalian RNF214 proteins for degradation. RNF214 proteins are poorly characterized, but our analyses suggest they are widely conserved among vertebrate organisms. We show that depletion of RNF214 levels in bat or human cells sensitizes these cells to arbovirus infection, revealing a new role for RNF214 proteins in antiviral defense. Our study demonstrates the utility of bacterial effectors as tools for identifying host immunity factors in mammalian hosts.

## Introduction

Arboviruses are transmitted by arthropod vectors to mammalian hosts. Whether arbovirus infection ensues after vector transmission depends upon the ability of the arbovirus to overcome mammalian antiviral defenses. Moreover, other mammalian species, such as bats, may serve as reservoirs for arboviruses that infect humans, such as togaviruses [e.g. Sindbis virus (SINV), Ross River virus (RRV), and O’nyong’nyong virus (ONNV)] [1–4]. Therefore, developing functional assays to identify mammalian factors relevant to arbovirus restriction is paramount.

We recently identified invertebrate antiviral factors by screening for bacterial effectors that enhance arbovirus replication in normally restrictive insect cells [5]. Bacterial effectors, which are proteins secreted by pathogenic bacteria into host cells, frequently act as immunomodulators. Consequently, these effectors serve as valuable tools for both inhibiting immune responses and identifying the key immunity factors they target [5]. Here, we show bacterial effectors can be used to identify arbovirus restriction factors in mammals such as bats and humans. By screening a library of 210 effectors, we identify dozens that enhance either togavirus or rhabdovirus replication in human or bat cells, suggesting effectors can inhibit virus- or host-specific restrictions. Moreover, three effectors enhanced togavirus and rhabdovirus replication in both host species. Characterization of one of these effectors, *Shigella flexneri*-encoded IpaH4, led us to identify mammalian RNF214 proteins as direct targets of IpaH4, and uncovered conserved roles for these host factors in restricting diverse viruses in bats and humans. Our work demonstrates that bacterial effector screening provides a functional platform for uncovering effector functions and mammalian antiviral machinery.

## Results

### Inhibition of host transcription enhances arbovirus replication in r06e bat cells

Previously, we showed the togavirus RRV and the rhabdovirus vesicular stomatitis virus (VSV) undergo abortive infections in *Lymantria dispar*-derived LD652 cells, but are rescued by actinomycin D (ActD) treatment, which inhibits cellular transcription [5, 6]. Thus, we wondered whether ActD could also enhance arbovirus replication in bat cells. If so, it would suggest mammalian host factors actively restrict their replication and we could then apply bacterial effector screens to identify mammalian factors restricting arbovirus replication.

*Rousettus aegyptiacus* (Egyptian fruit bat)-derived R06E cells were chosen due to genome availability for *R. aegyptiacus* [7], and evidence that *R. aegyptiacus* is a potential reservoir for toga- and rhabdoviruses [1, 2]; and other human pathogens, such as Marburg virus [8]. Thus, understanding *R. aegyptiacus* immunity may have implications for various viral diseases.

We infected R06E cells with GFP reporter arboviruses (RRV-GFP [9] and VSV^M51R^-GFP [6]) in the presence of various ActD concentrations. It is important to note VSV^M51R^-GFP is an attenuated strain with a reduced ability to block antiviral host responses [10–13]. Thus, we hypothesized it would be easier to detect enhanced replication with this mutant. ActD increased GFP signals during arbovirus infection in a dose-dependent manner, peaking at ~60- to 70-fold at the highest dose (10 nM) without affecting cell viability (S1A-C Fig). These findings suggest R06E cells express antiviral factors that restrict arbovirus replication.

### Bacterial effectors enhance arbovirus replication in bat and human cells

Certain bacterial effectors can rescue abortive arbovirus infections in lepidopteran cells [5]. To explore effector impact in mammalian cells, we adapted a 210-effector gene library [5] for lentivirus-based expression [14] and screened for effectors that enhanced RRV-GFP and/or VSV^M51R^-GFP replication in R06E cells. We also conducted screens in human U2OS cells to compare effector-mediated rescue in an additional mammalian species.

**Fig 1:**
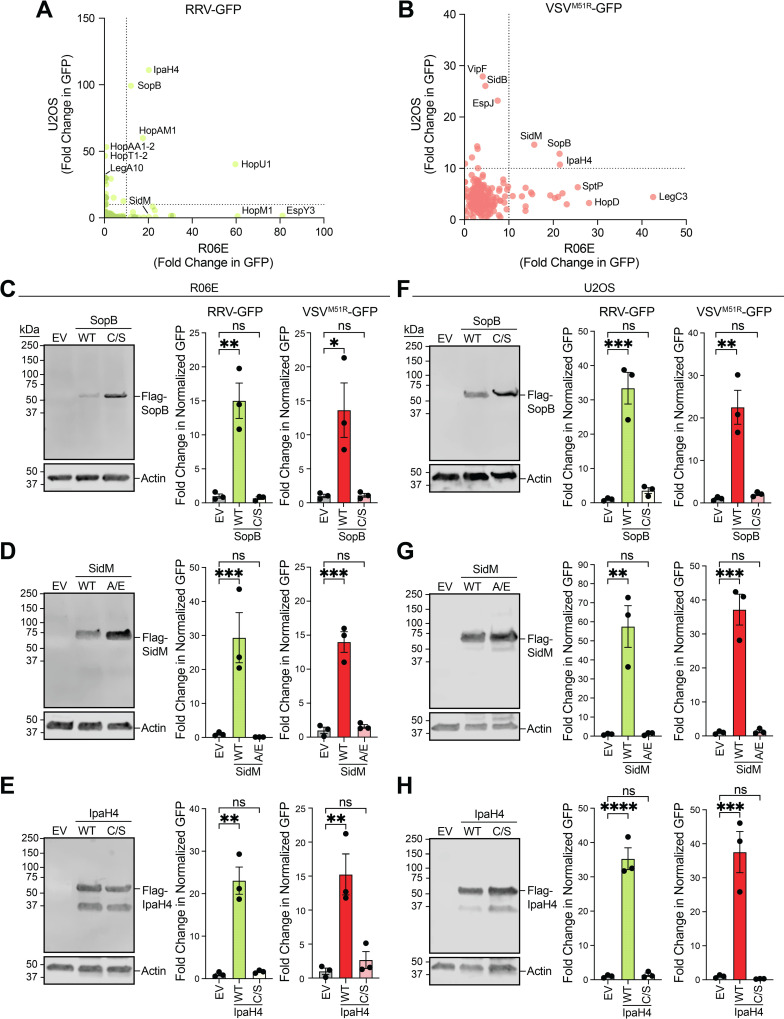
Identification of bacterial effectors that promote arbovirus replication in bat and human cells. A-B. Fold-change in viral GFP signals (normalized to CellTracker Blue signal) relative to cells transduced with lentivirus expressing firefly luciferase (LUC) (negative control) for RRV-GFP (**A**) and VSV^M51R^-GFP (**B**) screens 20 hpi. The cutoff for effectors to be scored as hits was a >10-fold change in over negative controls (cutoffs represented by dotted lines). Data points are means. **C-E.** Representative immunoblots of Flag-tagged bacterial effector expression in bat R06E cells 48 h post-transfection with pcDNA3.1 vectors. Additionally, fold-change in normalized viral GFP signal relative to cells transfected with empty vector (EV) is shown. Cells were stained 20 hpi with CellTracker Orange dye and imaged to calculate fold-change in normalized GFP signal over signals in EV treatments. Wild-type (WT) effectors are compared to their mutants (SopB^C420S^, SidM^A435E^, IpaH4^C339S^). **F-H.** Representative immunoblots and fold-change in normalized viral GFP signal for effectors experiments conducted as in **C-E** but in human U2OS cells. Quantitative data in **C-H** are means ± SEM; n=3. Statistical significance in graphs in **C-H** was determined with One-way ANOVA and Dunnett’s post-test; ns (not significant), *=P<0.05, **=*P*<0.01, ***=*P*<0.001, ****=*P*<0.0001.

R06E and U2OS cells were transduced with the effector library, infected with RRV-GFP or VSV^M51R^-GFP, and analyzed via fluorescence microscopy [5]. Effectors enhancing GFP signals >10-fold over control lentiviruses expressing firefly luciferase were considered “hits” (Fig 1A and 1B). Cytotoxic effectors (18 in bat and 4 in human cells) were excluded from further analyses (S1D and S1E Fig). RRV-GFP was enhanced by 15 or 23 effectors in R06E cells and U2OS cells, respectively. VSV^M51R^-GFP was enhanced by 18 in R06E cells and by 11 effectors in U2OS cells. Two effectors, SopB and IpaH4, enhanced both viruses in both cell types (Figs 1A, 1B, S2A, and S2B). While SidM did not enhance RRV-GFP in U2OS cells, it was a hit in all other screens (S2A and S2B Fig). Downstream analyses focused on SopB, SidM, and IpaH4, encoded by *Salmonella enterica* Typhimurium, *Legionella pneumophilia*, and *Shigella flexneri*, respectively. These results indicate that effectors from diverse bacterial pathogens can enhance arbovirus replication in mammalian cells.

### Effector activities are required for viral enhancement

To confirm expression and rescue functions of key effectors, we cloned Flag-tagged SopB, SidM, and IpaH4 into pcDNA3 and transfected cells for 48 h. SopB (inositol-phosphatase) [15], SidM (guanine nucleotide exchange factor) [16, 17], and IpaH4 (E3 ubiquitin ligase) [5] were also mutated to inactivate their known activities. Immunoblotting confirmed expression of both wild-type (WT) and mutant forms, but only WT effectors significantly increased arbovirus infection (Fig 1C-H), indicating their activity is essential for overcoming arbovirus restrictions in mammalian cells.

### IpaH4 E3 ubiquitin ligase activity is required for ubiquitination and degradation of host RNF214

**Fig 2:**
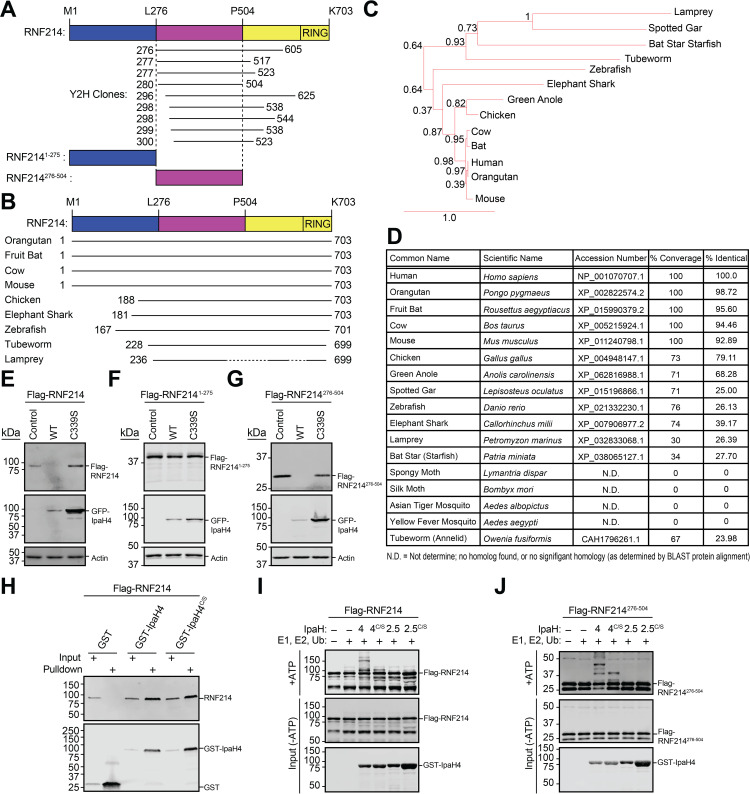
The central domain of the conserved host protein, RNF214, is sufficient for IpaH4-mediated ubiquitination and degradation. **A.** Schematic of human RNF214 with mapping of prey clones collectively identified in two yeast two-hybrid screens using IpaH4 bait [5]. Numbers indicate RNF214 a.a. encoded by each prey clone. **B.** Schematic displaying the relative conservation and length of RNF214 across indicated organisms. **C.** PhyloTv2-generated phylogenetic tree of RNF214 orthologs. **D.** Table of RNF214 orthologs in various vertebrate and invertebrate species generated via reciprocal BLAST analysis. **E.** Representative immunoblot of degradation assay using Flag-tagged RNF214 co-transfected into U2OS cells with GFP-IpaH4 (WT) or GFP-IpaH4^C339S^ (C339S) catalytic mutant expression vectors. **F-G.** Representative immunoblot of degradation assays using Flag-tagged RNF214 truncations, RNF214^1-275^ (**F**) or RNF214^276-504^ (**G**), co-transfected into U2OS cells with GFP-IpaH4 (WT) or GFP-IpaH4^C339S^ (C339S) vectors. **H.** Representative immunoblot of recombinant human Flag-RNF214 following GST pull-down after incubating with either GST, GST-IpaH4, or GST-IpaH4^C339S^. **I-J.** Representative immunoblot of *in vitro* ubiquitination assays showing direct IpaH4-mediated ubiquitination of purified, recombinant human Flag-RNF214 (**I**) and Flag-RNF214^276-504^ (**J**) proteins.

We previously identified *S. flexneri* IpaH4 as an E3 ubiquitin ligase [5]. Using yeast two-hybrid (Y2H) [5] and ubiquitin-activated interaction traps [18] with human prey, we found RNF214 to be the only putative target of IpaH4 that overlapped in both assays. RNF214 belongs to a large family of RING (really interesting new gene) finger (RNF) proteins, that typically function as E3 ubiquitin ligases [19]. Multiple Y2H clones targeting the RNF214 central region (a.a. 276-504) were detected (Fig 2A). We did not pursue RNF214 in our previous study examining IpaH4 targets in *L. dispar* cells, as RNF214 proteins are absent in moths and appear to be largely restricted to vertebrates, with notable exceptions like tubeworms and star fish (Fig 2B-D).

To assess whether IpaH4 promotes RNF214 degradation, we conducted degradation assays in U2OS cells. Flag-RNF214 levels decreased in the presence of WT IpaH4 but remained unchanged in the presence of a catalytically-inactive mutant (IpaH4^C339S^) (Fig 2E). Additionally, IpaH4 did not alter the levels of a N-terminal fragment (a.a. 1-275) (Fig 2F), but was sufficient to degrade a central (a.a. 276-504) RNF214 domain (Fig 2G), consistent with Y2H results. *In vitro,* Flag-RNF214 pulled down with GST-IpaH4 and GST-IpaH4^C339S^, but not with GST alone (Fig 2H). Additionally, IpaH4 ubiquitinated both full-length RNF214 and the central domain, while IpaH4^C339S^ and IpaH2.5 (another *S. flexneri* E3 ubiquitin ligase [20]), were unable to do so (Fig 2I and 2J). Thus, IpaH4 directly interacts with, and ubiquitinates, the central domain of RNF214 for degradation.

### RNF214 overexpression suppresses arbovirus infection of mammalian cells

**Fig 3:**
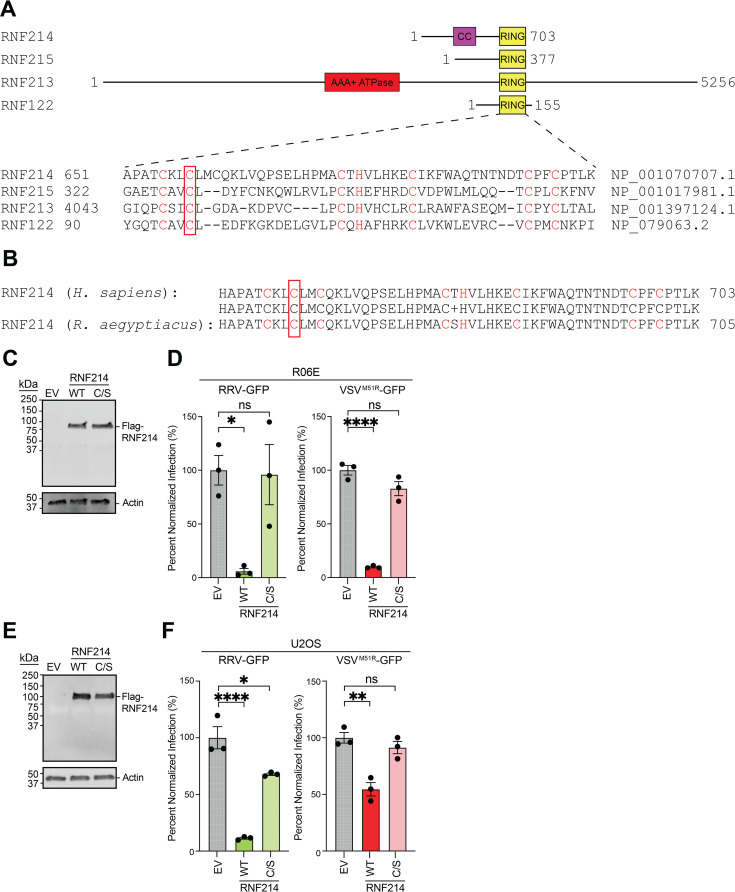
Overexpression of RNF214 suppresses arbovirus replication in bat and human cells **. A.** Cartoon protein maps and a.a. alignment of human RNF214 with other selected human RNF proteins. Residues in the characteristic RING domain “C4-H-C3” motif are highlighted in red, and the C658 residue targeted for substitution mutation is boxed. AAA+ ATPase domain in RNF213 is indicated [38]. **B.** a.a. alignment of human RNF214 with *R. aegyptiacus* RNF214. Residues are highlighted as described in **A**. **C.** Representative immunoblot of Flag-tagged RNF214 and RNF214^C658S^ (C/S) overexpression in R06E cells for 48 h. **D.** Percent of GFP signal relative to empty vector (EV) control after R06E cells overexpressing RNF214 constructs (WT or RNF214^C658S^) were infected with RRV-GFP (MOI=0.05) or VSV^M51R^-GFP (MOI=0.005) for 20 h. **E.** Representative immunoblot of Flag-tagged RNF214 constructs overexpressed in U2OS cells for 48 h. **F.** Percent of GFP signal relative to empty vector (EV) control in R06E cells overexpressing Flag-RNF214 constructs (WT or RNF214^C658S^) and infected with RRV-GFP (MOI=0.05) or VSV^M51R^-GFP (MOI=0.005) for 20 h. Data are means ± SEM; n=3. Statistical significance was determined with One-way ANOVA and Dunnett’s post-test; ns (not significant), *=P<0.05, **=*P*<0.01, ***=*P*<0.001, ****=*P*<0.0001.

To determine if RNF214 mediates arbovirus restriction, we overexpressed Flag-RNF214 in bat and human cells and then infected them with arboviruses. Bioinformatic alignment of the RNF domain identified C658 as a conserved residue likely essential for E3 ligase activity (Fig 3A and 3B) [21]. Overexpression of WT Flag-RNF214 significantly reduced RRV-GFP and VSV^M51R^-GFP replication in both cell types. In contrast, the putative catalytic mutant (Flag-RNF214^C658S^) did not inhibit arbovirus replication in bat cells or VSV^M51R^-GFP replication in U2OS cells, although it slightly inhibited RRV-GFP in U2OS cells (Fig 3C-F). These data suggest RNF214 largely suppresses arbovirus replication in mammalian cells via its putative ubiquitin ligase activity.

### RNF214 depletion enhances virus replication but does not affect cell death or interferon-stimulated gene expression

**Fig 4:**
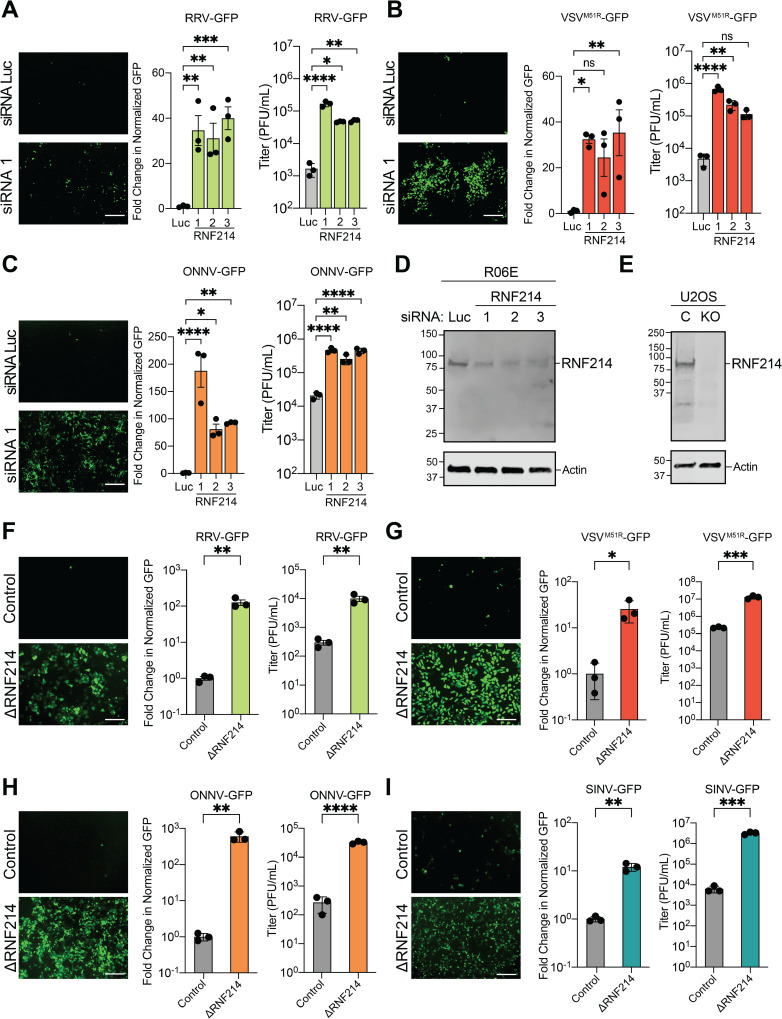
Depletion of RNF214 enhances arbovirus replication in bat and human cells. **A.** Representative fluorescence microscopy images (GFP channel) of R06E cells transfected with firefly luciferase (Luc)- or RNF214-targeting siRNAs. 48 h post-knockdown, cells were infected with RRV-GFP (MOI=0.001) for 20 h. Cells were then stained with CellTracker Orange, imaged, and fold-change in GFP signal compared to the Luc control was calculated. Supernatants were also collected to assess viral titers by plaque assay. **B-C.** Similar experiments were performed as in A but for VSV^M51R^-GFP (MOI=0.0001) (**B**) and ONNV-GFP (MOI=0.001) (**C**). **D.** Representative immunoblot of endogenous bat RNF214 levels following siRNA knockdown in R06E cells for 48 h. **E.** Representative immunoblot of endogenous human RNF214 levels in U2OS cells transduced with control lentivirus (**C**) or lentivirus expressing guide RNAs targeting RNF214 (KO). **F.** Representative fluorescence microscopy images (GFP channel) in control or U2OS^ΔRNF214^ cells infected with RRV-GFP (MOI=0.001) for 20 h. Cells were stained with CellTracker Orange, imaged, and fold-change in GFP signal compared to control U2OS cell infections was calculated. Supernatants were also collected to assess viral titers by plaque assay. **G-I.** Similar experiments were performed as in **F** but for VSV^M51R^-GFP (MOI=0.0001) (**G**), ONNV-GFP (MOI=0.001) (**H**) and SINV-GFP (MOI=0.001) (**I**). Data are means ± SEM; n=3. Statistical significance for **A-C** was determined with One-way ANOVA and Dunnett’s post-test, and with unpaired Student’s t-test for **F-I**; ns (not significant), *=P<0.05, **=*P*<0.01, ***=*P*<0.001, ****=*P*<0.0001. Scalebars indicate 200 μm on all microscopy images.

To further assess the role of RNF214 in virus restriction, we knocked down RNF214 in R06E and U2OS cells and challenged them with arboviruses. RRV-GFP and VSV^M51R^-GFP replication increased in at least 2/3 RNF214 siRNA treatments, and a third togavirus (ONNV-GFP [5]) also showed increased replication with RNF214 knockdown. Importantly, we confirmed knockdown in both cell types (Figs 4A-D and S3A-C). Similarly, knockout of RNF214 in U2OS cells (U2OS^ΔRNF214^) led to 10-1000-fold increases in GFP signal and viral titers for RRV-GFP, VSV^M51R^-GFP, ONNV-GFP, and SINV-GFP [6] (Fig 4E-I).

Importantly, we found no differences in cell growth rate or death between control and U2OS^ΔRNF214^ cells (S3D-F Fig). Additionally, RNF214 knockout had no effect on interferon (IFN) beta or IFN-stimulated gene expression (S3G-J Fig). These data suggest RNF214 may play an antiviral role independent of cell death and Type I IFN.

We next tested a diverse panel of viruses for enhanced replication in U2OS^ΔRNF214^ cells. We did not observe enhanced replication for the DNA viruses, vaccinia virus (VACV-FL-GFP) [22] and herpes simplex virus 1 (HSV-1-GFP) [23] (S4A and S4B Fig). In contrast, WT VSV-GFP replication was moderately enhanced (S4C Fig), suggesting that even non-attenuated VSV strains are restricted by RNF214. Other negative-sense ssRNA viruses, such as the paramyxoviruses, human parainfluenza virus 1 (HPIV1-GFP) [24] and Sendai virus (SeV-GFP) [23] either exhibited no change or minor increases in viral replication (S4D and S4E Fig). A similar minor enhancement was observed with the positive-sense ssRNA flavivirus, West Nile virus (WNV-GFP [14]) (S4F Fig). Interestingly, Venezuelan equine encephalitis virus (VEEV-GFP [25, 26]) showed a ~15-fold increase in titer in U2OS^ΔRNF214^ cells (S4G Fig), consistent with our observations with other togaviruses (Fig 4F and 4H-I). In summary, these results suggest that RNF214 restricts specific ssRNA viruses but not DNA viruses (S4H Fig).

## Discussion

Here, we used bacterial effectors to break arbovirus restriction and identify antiviral factors in mammalian cells. We identified 30 effectors that enhance arbovirus infection in bat cells and 29 in human cells. Among these, SopB, SidM, and IpaH4 promoted togavirus and rhabdovirus replication in both species.

Our investigation focused on the IpaH4 E3 ubiquitin ligase from *S. flexneri*, which we found to ubiquitinate and degrade host RNF214. Overexpression of RNF214 suppressed, while its depletion enhanced, arbovirus infection in bat and human cells, indicating RNF214 as a potent antiviral factor. Screening across 11 viruses, we observed RNF214 to specifically restrict ssRNA viruses. This suggests RNF214 belongs to an ever-expanding group of E3 ubiquitin ligases involved in intracellular pathogen restriction [19,27,28].

RNF proteins represent the largest E3 ubiquitin ligase family with over 340 members in humans alone [19]. Recent studies suggest emerging and important roles for RNF proteins in regulating or mediating innate immune responses to pathogens. For example, RNF5, RNF122, and RNF215 have been shown to negatively regulate NF-κB-, RIG-I-, or STING-mediated IFN responses during viral infection [29–31]. In contrast, other RNF proteins, such as RNF213, actively restrict diverse pathogens including viruses, bacteria, and intracellular parasites [32–35]. Interestingly, RNF214 has recently been shown to influence the Hippo pathway [36], which can regulate cellular proliferation and death, and this pathway has been shown to be targeted by viruses such as SARS-CoV-2 [37]. However, our results suggest that cell proliferation and death are unchanged in RNF214 knockout cells and thus cannot explain RNF214 antiviral activity. Moreover, because RNF214 did not affect IFN-β or IFN-stimulated gene expression, RNF214 may not exert its antiviral activity by regulating IFN responses. These observations are consistent with the fact that RNF214 specifically restricted ssRNA viruses because, if RNF214 regulated cell death or IFN responses, it would likely inhibit a broader array of viruses. Further studies are needed to confirm the E3 ligase activity of RNF214 *in vitro* and define its antiviral mechanism.

## Materials and methods

### Cell lines and cell culture

U2OS and BSC-40 cells were cultured as previously described [23]. R06E cells were cultured in DMEM:F12 supplemented with 10% FBS and 1% antibiotic/antimycotic (Gibco; S2 Table).

### Viruses

Stock preparation, culture of recombinant viruses, and titration by fluorescent foci/plaque assay on BSC-40 cells was performed as previously described [5, 6].

### Cell viability assay

Cell viability was measured using a LDH assay (Invitrogen; S2 Table) as described [5] 48 h post-transduction of cells with lentiviruses.

Additional details regarding Materials and Methods and key reagents and resources can be found in S1 Text and S2 Table, respectively.

## Supporting information

S1 Text Supplementary experimental details.
(DOCX)

S1 Table Bacterial effector library screening results and cell viability data.
(XLSX)

S2 Table Key resources and reagents.
(XLSX)

S1 Fig **Effect of ActD treatment on arbovirus infection of mammalian cells and impact of effector expression on mammalian cell viability. A.** Representative fluorescence microscopy images (GFP channel) of R06E cells infected with RRV-GFP (MOI=0.001) or VSV^M51R^-GFP (MOI=0.0001) and treated with either DMSO or 10 nM ActD for 20 h. Scalebars indicate 200 μm. **B.** Fold-change in GFP reporter readout following 20 h infection in the presence of the indicated doses of ActD, normalized to DMSO control. **C.** Results of LDH-based cytotoxicity assays in R06E cells. Absorbance at 490 nm is plotted for supernatant collected from R06E cells treated for 20 h with increasing doses of ActD. Positive (+) control supplied by the manufacturer, as well as cells lysed with manufacturer 10X lysis buffer (lysed cells) are also plotted for reference. **D.** Results of LDH-based cytotoxicity assays in R06E cells transduced with effector library for 48 h. LDH values that were significantly higher (P<0.05) from Luc-transduced control cells were considered toxic effectors (red bars). **E.** Results of LDH-based cytotoxicity assays in U2OS cells transduced with effector library for 48 h as in **D**. Red bars indicate toxic effectors. Data are means ± SEM; n=3. Statistical significance for **B-C** was determined with One-way ANOVA and Dunnett’s post-test; ns (not significant), *=P<0.05, **=*P*<0.01, ***=*P*<0.001, ****=*P*<0.0001.
(TIF)

S2 Fig **Specific bacterial effectors enhance arbovirus replication in bat and human cells. A.** Summary of bacterial effectors that rescued RRV-GFP replication in bat or human cells, or both cell types. Green blocks indicate the effector enhanced RRV-GFP in the cell line indicated in the column header. Effectors are listed from high-to-low based on their fold-change in GFP signal over controls. The bacterium encoding each effector is noted to the right: *Shigella flexneri* (*S. flexneri*), *Pseudomonas syringae* (*P. syringae*), *Salmonella enterica* (*S. enterica*), *Legionella pneumophila* (*L. pneumo*.) *Enterohemorrhagic Escherichia coli* 0157:H7 (*EHEC*), or *Bartonella henselae* (*B. henselae*). **B.** Summary of bacterial effector proteins that enhanced VSV^M51R^-GFP replication in bat or human cells, or both cell types. The complete list of effectors screened and the raw results of the screens can be found in **S1 Table**.
(TIF)

S3 Fig **RNF214 knockdown enhances arbovirus replication in human U2OS cells and RNF214 knockout does not affect cell viability or Type I IFN responses. A.** Representative immunoblot of RNF214 levels in U2OS cells transfected with indicated siRNAs for 48 h. **B.** Fold-change in GFP reporter readout following RRV-GFP infection of U2OS cells after indicated knockdowns. Cells were infected (MOI=0.001) 48 h post-siRNA transfection for 20 h, stained with CellTracker Orange, and imaged to determine the fold-change in GFP signal compared to Luc siRNA (control) treatments. **C.** Fold-change in GFP reporter readout following VSV^M51R^-GFP infection of U2OS cells after indicated knockdowns. Cells were infected (MOI=0.0001) 48 h post-siRNA transfection for 20 h, stained with CellTracker Orange, and imaged to determine the fold-change in GFP signal compared to Luc siRNA (control) treatments. **D.** Cell counts of control or U2OS^ΔRNF214^ cells over a 72 h time course. **E-F.** Results of cytotoxicity assays in control or U2OS^ΔRNF214^ cells infected at increasing MOIs of VSV^M51R^-GFP for 20 h. Following infection, supernatants were collected for LDH-based cytotoxicity assays (**E**) and cells were lysed for CellTiter-Glo-based cell viability assays (**F**). “Lysed” refers to cells lysed in lysis buffer to serve as a positive control for cell death. **G-I.** Fold-change in *IFNB1* (**G**), *IFIT1* (**H**), and *RSAD2* (Viperin) (**I**) transcript levels (compared to mock-infected control cell levels) in control or U2OS^ΔRNF214^ cells infected with VSV^M51R^-GFP (MOI=0.0001) for 20 h. *IFNB1* encodes IFN-β whereas *IFIT1* and *RSAD2* are IFN-stimulated genes [39, 40]. **J.** Representative immunoblot of endogenous human IFIT3 and ISG15 (which are encoded by IFN-stimulated genes [40]) levels following treatment of control or U2OS^ΔRNF214^ cells with increasing doses of recombinant IFN-β. Data in **B-I** are means ± SEM; n=3. Statistical significance for **B,C** and the multiple comparisons of **G-I** was determined with One-way ANOVA and Dunnett’s post-test; ns (not significant), *=P<0.05, **=*P*<0.01, ***=*P*<0.001, ****=*P*<0.0001. Statistical significance for **E-I** was determined with unpaired Student’s t-test; ns (not significant), *=P<0.05, **=*P*<0.01, ***=*P*<0.001, ****=*P*<0.0001.
(TIF)

S4 Fig **Assessing the impact of RNF214 knockout on the replication of diverse viral families. A-B.** Control or U2OS^ΔRNF214^ cells infected with the indicated reporter poxvirus (VACV-FL-GFP; MOI=0.01) or herpesvirus (HSV-1-GFP; MOI=0.01). Following infection, cells were stained with CellTracker Orange and imaged for fold-change in GFP reporter readout compared to control cells. Titers are also shown from these collected cultures. **C-G.** Similar experiments were performed as in **A-B** but with VSV-GFP (MOI=0.0001) (**C**), the paramyxoviruses: HPIV1-GFP (MOI=0.001) (**D**) and SeV-GFP (MOI=0.001) (**E**), the flavivirus, WNV-GFP (MOI=0.001) (**F**) and the togavirus, VEEV-GFP (MOI=0.001) (**G**). **H.** Table summarizing results for the various viral families tested in this figure. Data for **A-G** are means ± SEM; n=3. Statistical significance was determined with unpaired Student’s t-test; ns (not significant), *=P<0.05, **=*P*<0.01, ***=*P*<0.001, ****=*P*<0.0001.
(TIF)
