# Supplementary material for: Bacterial effector screening reveals RNF214 as a virus restriction factor in mammals
Source: PLoS Pathog. 2025 Apr 22;21(4):e1013035. doi: 10.1371/journal.ppat.1013035 (PMC12013929; doi:10.1371/journal.ppat.1013035)
Supplement: S1 Text — Supplementary experimental details. (DOCX) [file ppat.1013035.s001.docx]

**Materials and Methods**

*Cell Lines and Cell Culture*

Mammalian cell lines were maintained at 37°C in 5% CO_2_ atmosphere. U2OS cells were cultured in DMEM supplemented with 10% FBS containing 1% non-essential amino acids (NEAA), 1% L-glutamine, and 1% antibiotic/antimycotic (Gibco; S2 **Table**). R06E cells were cultured in DMEM:F12 supplemented with 10% FBS and 1% antibiotic/antimycotic (Gibco). BSC-40 cells were cultured in MEM supplemented with 5% FBS containing 1% non-essential amino acids (NEAA), 1% L-glutamine, and 1% antibiotic/antimycotic (Gibco; S2 **Table**).

*Viruses*

Stock preparation, culture of recombinant viruses, and titration by fluorescent foci/plaque assay on BSC-40 cells was performed as previously described [1, 2]. Viral innocula were incubated with cells for 1 h in serum free media before the addition of complete media for the remainder of the infection. Where indicated, complete media containing ActD (Fisher Bioreagents; S2 **Table**) at the indicated dose was added for the remainder of the infection.

*Cell Viability Assays*

Cell viability was measured using a CyQUANT^TM^ LDH Cytotoxicity assay (Invitrogen; S2 **Table)** as previous described [2] 48 h post-transduction of cells with the effector expression library. CellTiter-Glo (Promega; S2 **Table**) viability experiments were conducted on U2OS and U2OS^ΔRNF214^ cells 20 h post-infection according to the manufacturer’s protocol. The viability for each cell line was normalized to a mock-infected U2OS control sample.

*Plasmid Constructs for Mammalian Cell Expression*

The 210 bacterial effector protein coding sequences was generated in pENTR/D as previously described [3]. Briefly, PCR-amplified bacterial genomic DNA sequences were cloned into pENTR/D-TOPO (Invitrogen) using topoisomerase I. Source organisms were *Pseudomonas syringe* pv. Tomato (ATCC BAA-871D-5), *Legionella pneumophila* Philadelphia-1 (ATCC 33152D-5), *Salmonella Typhimurium* LT2 (gift of Jack Dixon, University of California San Diego), EHEC H7:O157 (gift of Vanessa Sperandio, University of Wisconsin-Madison), *Shigella flexneri* M90T (gift of Jack Dixon, University of California San Diego), and *Bartonella henselae* Houston-1 (gift of Alexei Savchenko, University of Toronto). For expression in mammalian cells, the library was cloned into the lentiviral expression vector pTRIP-CMV-IVSb-IRES-TagRFP [4] using Gateway LR Clonase II (Invitrogen; S2 **Table**).

RNF214 was Gibson cloned from pENTR221 (DNASU HsCD00513497) into pFLAG-CMV-6b, imparting an N-terminal Flag tag. Flag-tagged RNF214 fragments were also generated in pFLAG-CMV-6b by Gibson cloning. For bacterial expression, sequence encoding Flag-RNF214 or only the 276-504 a.a. RNF214 fragment were Gibson cloned into pProEX-HTb (Invitrogen), imparting a 6xHis tag at the N-terminus upstream of the Flag sequence.

N-terminal Flag-tagged versions of SopB (WFG56166.1), SidM (YP_096471.1), and C-terminal Flag-tagged IpaH4 (EID62426.1) wild-type and point mutants were generated as previously described and cloned into pcDNA3.1 for expression in mammalian cells [2].

*General Transfection Protocols*

Unless otherwise stated, 100,000 cells were plated into 24-well dishes, transfected with 500 ng of expression vectors for 48 h prior to manipulation (e.g. protein extraction or virus infection). For U2OS cell expression of the pcDNA3.1 constructs, 500 ng of vector was mixed with 100 μL of OptiMEM media and 1.5 μL of Lipofectamine 2000 Reagent (S2 **Table**). This mixture was incubated at room temperature for 20 min, and media on the cells was changed to 500μL OptiMEM, then the transfection mixture was added dropwise to wells, incubated overnight and media was then changed to complete DMEM ~16 h post-transfection.

For R06E transfection experiments, as well as HEK293T transfection, 500 ng of vector was mixed with 50 μL of Opti-MEM and 1 μL of FuGENE HD Transfection Reagent (Promega; **S2 Table**). This mixture was incubated at room temperature for 20 min before adding dropwise to the well. Cells were then incubated for 48 h prior to further manipulation or infection.

Transient siRNA-mediated knockdown was achieved by reverse transfection of R06E cells with 8 pmol siRNA and 1.5 μL of FuGENE HD Reagent according to the manufacturer’s protocol. The same protocol was used for U2OS cells except Lipofectamine 2000 was used as the transfection reagent according to the manufacturer’s protocol. Cells were transfected for 48 h and then either subjected to protein extraction and immunoblotting or were subjected to GFP reporter virus infection to assess replication phenotypes.

*qRT-PCR*

RNA was extracted from U2OS and U2OS^ΔRNF214^ cells infected with VSV^M51R^-GFP at an MOI=0.001 for 20 h according to manufacturer’s protocol using RNeasy Plus Universal Mini Kit (Qiagen; S2 **Table**). RNA input was then normalized to 50 ng/well before qRT-PCR was performed with QuantiNova SYBR Green PCR Kit as previously outlined and according to the manufacturer’s protocol (Qiagen; S2 **Table**) [5]. Ct values were converted into relative gene expression levels compared to control (mock-infected conditions in control cells) using the ΔΔCt method [6]. Primers directed against the housekeeping gene GAPDH, were used to normalize IFNB1 and IFN-stimulated gene expression, IFIT1 and RSAD2 (Viperin). Melt curves were generated for all experiments by ramping up the temperature 1°C/minute from 60°C to 95°C. Only a single melt curve peak was observed for all primer sets.

*IFN Treatment*

Both U2OS and U2OS^ΔRNF214^ cells were plated at 100,000 cells per well into 24-well dishes, and treated with increasing doses of recombinant human IFN-β for 20 h (Pestka Biomedical Laboratories; S2 **Table**). Cells were then lysed in 100 μL RIPA buffer containing 100 μM PMSF and protease inhibitor before immunoblotting.

*Immunoblotting*

Immunoblotting was conducted as previously described with commercial primary antibodies and secondary antibodies conjugated with infrared dyes and a Li-Cor Odyssey scanner [2].

*Bacterial Effector Screens and Fluorescence Microscopy*

Cells were seeded into 96-well clear bottom dishes, transduced with the lentivirus effector library in quadruplicate for 48 h, and then challenged with indicated GFP reporter viruses. At the indicated times post-infection, cells were stained as previously described [2] with CellTracker Dye, fixed with PFA, and cells were imaged by the UT Southwestern Medical Center High-Throughput Screening Core using an INCell Analyzer 6000 (Molecular Devices) scope equipped with 405, 488, and 561 nm lasers using a using a 10x objective. Four images were taken/well so that fluorescence signals/well resulted from an average of these four images. Signals across all 4 replicate wells for each effector treatment were then used to determine GFP reporter virus infection levels. Image analysis was conducted using Fiji 2.14.0 (NIH) to quantify the percent area of each field of view containing GFP signal and these signals were normalized to CellTracker Dye signal to account for cell number [2]. Finally, normalized GFP signals for each effector treatment were plotted as a fold change in GFP signal relative to cells transduced with lentivirus vector expressing firefly luciferase (LUC) (negative control). Validation assays to confirm virus-enhancing phenotypes after transfection of pcDNA3.1 expression plasmids encoding Flag-tagged effector constructs were conducted and analyzed in a similar manner except empty pcDNA3.1 vector was used as a negative control treatment.

*RNF214 Degradation Assay*

~75,000 cells were co-transfected with 150 ng target pcDNA3.1 vectors encoding full-length Flag-RNF214 (or RNF214 fragments) and 350 ng of pEGFP-C2 encoding either GFP, GFP-IpaH4, or GFP-IpaH4^C339S^ [2] using 1.5 μL of X-tremeGENE 9 and 50 μL of OptiMEM (Sigma; S2 **Table**). After 24 h, cells were harvested directly in 1X Laemmeli buffer containing β-mercaptoethanol. Protein extracts were then subjected to SDS-PAGE and subsequent immunoblotting with indicated antibodies.

*Protein Purification*

For human Flag-RNF214 full length or fragment expression *E. coli*BL21 cells were transformed with pProEX-HTb-6xHis-FLAG-RNF214 or pProEX-HTb-6xHis-FLAG-RNF214^276-504^ vectors and spread onto LB agar plates containing 100 µg/mL ampicillin. A colony was picked and grown in a 30 mL LB culture with 100 µg/mL ampicillin overnight at 37°C with shaking. The following day, this culture was added to 1 L of LB with ampicillin and grown at 37°C with shaking to an OD600 of 0.8. Protein expression was then induced by the addition of 0.5 mM IPTG and the culture was grown overnight at 18°C with shaking. Bacteria were pelleted at 4000xg for 20 min (Beckman Avanti J-25) and resuspended in 30 mL of purification buffer (20 mM HEPES, 150 mM NaCl, 1 mM TCEP, and 1X SigmaFAST protease inhibitor (Sigma; S2 **Table**), pH=7.5). Lysate was clarified by centrifugation at 10,000xg for 15 min. The fusion protein was then affinity-purified over an Econo-Pac chromatography column (Biorad; S2 **Table**) using TALON Metal Affinity Resin (Takara 635502) and eluted in purification buffer containing 500 mM imidazole. The protein was dialyzed using a 10,000 MWCO Slide-A-Lyzer dialysis cassette (Thermo; S2 **Table**) in 1 L of purification buffer without imidazole. Protein was combined with glycerol at 20% and stored at -80°C. Recombinant GST-IpaH4 for *in vitro* ubiquitination experiments was expressed and purified as previously described [2].

*In Vitro Ubiquitination Assay*

*In vitro* ubiquitination reactions were performed as previously described [2] with minor modifications. Briefly, reactions were performed in 50 mM HEPES pH 7.5, 150 mM NaCl, 20 mM MgCl_2_, and 10 mM ATP in a volume of 30 µL. The following recombinant proteins were added: 1 µM UbE1 (E1), 5 µM UbcH5b (E2), 5 µM GST-IpaH or GST-IpaH4^C339S^, 50 µM ubiquitin, and 5 µM 6xHis-FLAG-tagged RNF214 or its truncated versions. ATP was added last to initiate reactions, which were then incubated for 2 h at 30°C before the addition of 30 µL 2X Laemmli buffer containing β-mercaptoethanol. Samples were then boiled for 10 min at 95°C, subjected to SDS-PAGE, and immunoblotting.

*In vitro GST Pull-down Assay*

*In vitro* GST pull-downs were performed in 50 mM Tris-HCl (pH7.6), 50 mM NaCl, 1 mM EDTA, 0.5% NP40 at a volume of 1,000 µL. The following recombinant proteins were added: 5 µM 6XHis-Flag-RNF214, with either 1 µM GST, GST-IpaH, or GST-IpaH4^C339S^. Input was collected before mixtures were incubated for 6 h at 4°C, then 30 µL glutathione sepharose beads were added for 2 h (GE Healthcare Life Sciences; S2 **Table**). Samples were washed three times with 1 mL of the above mentioned pull-down buffer for 5 min at RT before the addition of 50 µL pull-down buffer and 50 µL 2X Laemmli buffer containing β-mercaptoethanol. Samples were then boiled for 5 min and subjected to SDS-PAGE, and immunoblotting.

*Lentivirus Production*

HEK293T cells were plated in poly-D-lysine coated 6-well plates at 400,000 cells/well in 2 mL/well to yield a confluency of 50% the following day. Media was changed to 1.5 mL/well DMEM containing 3% FBS and 1% NEAA. Cells were then transfected with 200 ng/well pCMV-VSVG, 800 ng/well pCMV-Gag/Pol, and 1000 ng/well pTRIP-CMV-Effector-IVSb-IRES-TagRFP using X-tremeGENE 9 (Sigma; S2 **Table**) and OptiMEM (Thermo; S2 **Table**). Following 6 h of incubation, media was changed to 1.5 mL/well DMEM with 3% FBS and 1% NEAA. At 48 h post-media change, the supernatant was collected and replaced with fresh media. After an additional 24 h, the supernatant was again collected. Supernatants from both time points were pooled, centrifuged at 3000xg for 5 min to remove cell debris, and combined with HEPES to a concentration of 20 mM and polybrene to a concentration of 4 µg/mL. Lentivirus stocks were then arrayed in v-bottom 96-well plates and stored at -80°C.

*Statistical Analyses*

Graphs were presented as mean values ± SEM with individual data points shown. At least three independent experiments were conducted for all quantitative experiments shown where statistical analyses were applied. All statistical analyses were performed with Prism software v10.0.2 (GraphPad) and statistical tests used are indicated in respective figure legends. Statistical significance (*P*<0.05) between compared groups is indicated in figures as either: ns (not significant), *=P<0.05, **=*P*<0.01, ***=*P*<0.001, ****=*P*<0.0001.

**Supplementary References**

1. Gammon DB, Duraffour S, Rozelle DK, Hehnly H, Sharma R, Sparks ME, et al. A single vertebrate DNA virus protein disarms invertebrate immunity to RNA virus infection. Elife. 2014;3. Epub 2014/06/27. doi: 10.7554/eLife.02910. PubMed PMID: 24966209; PubMed Central PMCID: PMCPMC4112549.

2. Embry A, Baggett NS, Heisler DB, White A, de Jong MF, Kocsis BL, et al. Exploiting bacterial effector proteins to uncover evolutionarily conserved antiviral host machinery. PLoS Pathog. 2024;20(5):e1012010. Epub 20240516. doi: 10.1371/journal.ppat.1012010. PubMed PMID: 38753575; PubMed Central PMCID: PMCPMC11098378.

3. Weigele BA, Orchard RC, Jimenez A, Cox GW, Alto NM. A systematic exploration of the interactions between bacterial effector proteins and host cell membranes. Nat Commun. 2017;8(1):532. Epub 2017/09/16. doi: 10.1038/s41467-017-00700-7. PubMed PMID: 28912547; PubMed Central PMCID: PMCPMC5599653.

4. Richardson RB, Ohlson MB, Eitson JL, Kumar A, McDougal MB, Boys IN, et al. A CRISPR screen identifies IFI6 as an ER-resident interferon effector that blocks flavivirus replication. Nat Microbiol. 2018;3(11):1214-23. Epub 20180917. doi: 10.1038/s41564-018-0244-1. PubMed PMID: 30224801; PubMed Central PMCID: PMCPMC6202210.

5. Boys IN, Mar KB, Schoggins JW. Functional-genomic analysis reveals intraspecies diversification of antiviral receptor transporter proteins in Xenopus laevis. PLoS Genet. 2021;17(5):e1009578. Epub 20210520. doi: 10.1371/journal.pgen.1009578. PubMed PMID: 34014925; PubMed Central PMCID: PMCPMC8172065.

6. Livak KJ, Schmittgen TD. Analysis of relative gene expression data using real-time quantitative PCR and the 2(-Delta Delta C(T)) Method. Methods. 2001;25(4):402-8. doi: 10.1006/meth.2001.1262. PubMed PMID: 11846609.
