# Supplementary figures and images for: Bacterial effector screening reveals RNF214 as a virus restriction factor in mammals
Source: PLoS Pathog. 2025 Apr 22;21(4):e1013035. doi: 10.1371/journal.ppat.1013035 (PMC12013929; doi:10.1371/journal.ppat.1013035)

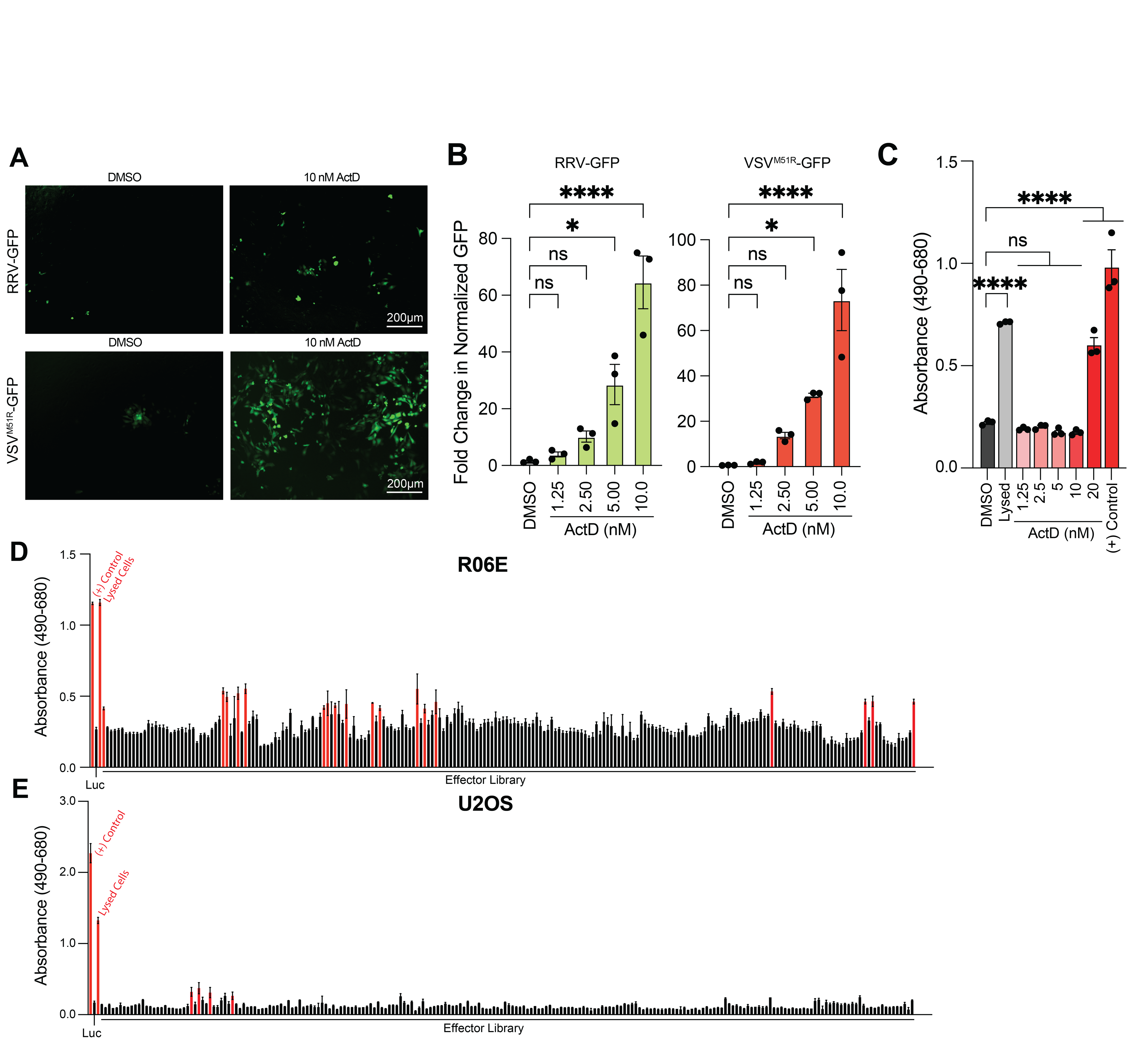

Supplement: S1 Fig — Effect of ActD treatment on arbovirus infection of mammalian cells and impact of effector expression on mammalian cell viability. A. Representative fluorescence microscopy images (GFP channel) of R06E cells infected with RRV-GFP (MOI=0.001) or VSVM51R-GFP (MOI=0.0001) and treated with either DMSO or 10 nM ActD for 20 h. Scalebars indicate 200 μm. B. Fold-change in GFP reporter readout following 20 h infection in the presence of the indicated doses of ActD, normalized to DMSO control. C. Results of LDH-based cytotoxicity assays in R06E cells. Absorbance at 490 nm is plotted for supernatant collected from R06E cells treated for 20 h with increasing doses of ActD. Positive (+) control supplied by the manufacturer, as well as cells lysed with manufacturer 10X lysis buffer (lysed cells) are also plotted for reference. D. Results of LDH-based cytotoxicity assays in R06E cells transduced with effector library for 48 h. LDH values that were significantly higher (P<0.05) from Luc-transduced control cells were considered toxic effectors (red bars). E. Results of LDH-based cytotoxicity assays in U2OS cells transduced with effector library for 48 h as in D. Red bars indicate toxic effectors. Data are means ± SEM; n=3. Statistical significance for B-C was determined with One-way ANOVA and Dunnett’s post-test; ns (not significant), *=P<0.05, **=P<0.01, ***=P<0.001, ****=P<0.0001. (TIF) [file ppat.1013035.s004.tif]

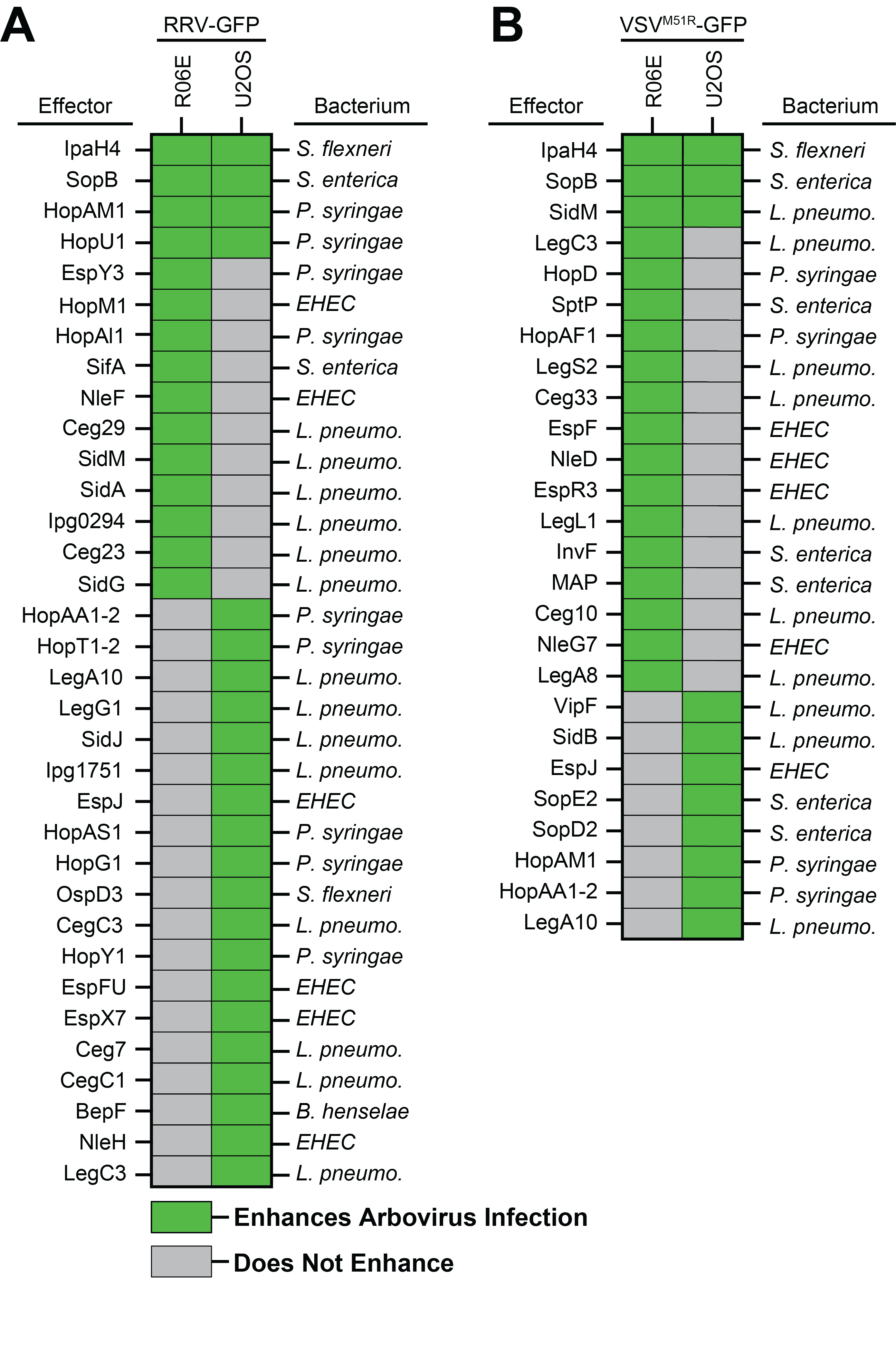

Supplement: S2 Fig — Specific bacterial effectors enhance arbovirus replication in bat and human cells. A. Summary of bacterial effectors that rescued RRV-GFP replication in bat or human cells, or both cell types. Green blocks indicate the effector enhanced RRV-GFP in the cell line indicated in the column header. Effectors are listed from high-to-low based on their fold-change in GFP signal over controls. The bacterium encoding each effector is noted to the right: Shigella flexneri (S. flexneri), Pseudomonas syringae (P. syringae), Salmonella enterica (S. enterica), Legionella pneumophila (L. pneumo.) Enterohemorrhagic Escherichia coli 0157:H7 (EHEC), or Bartonella henselae (B. henselae). B. Summary of bacterial effector proteins that enhanced VSVM51R-GFP replication in bat or human cells, or both cell types. The complete list of effectors screened and the raw results of the screens can be found in S1 Table. (TIF) [file ppat.1013035.s005.tif]

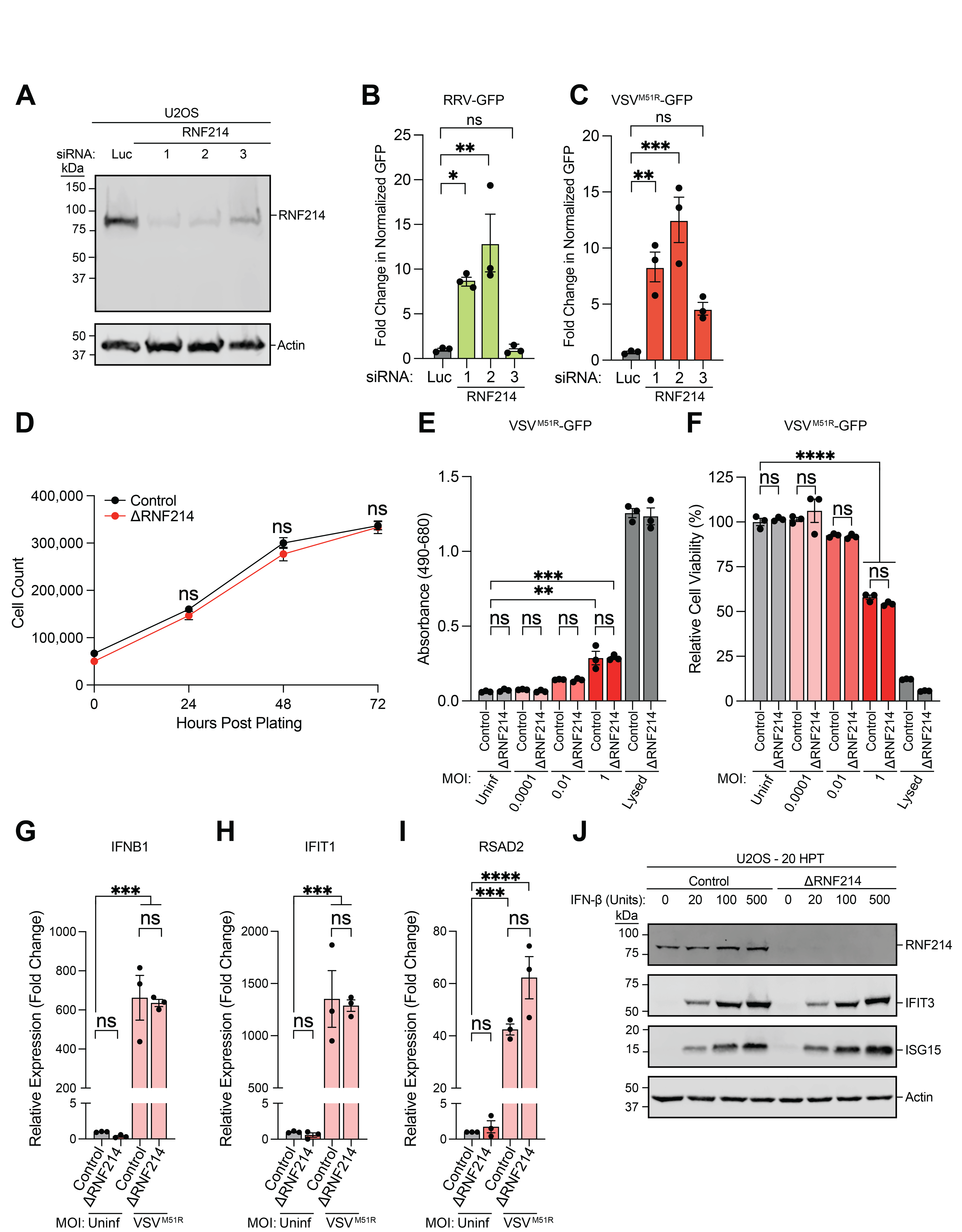

Supplement: S3 Fig — RNF214 knockdown enhances arbovirus replication in human U2OS cells and RNF214 knockout does not affect cell viability or Type I IFN responses. A. Representative immunoblot of RNF214 levels in U2OS cells transfected with indicated siRNAs for 48 h. B. Fold-change in GFP reporter readout following RRV-GFP infection of U2OS cells after indicated knockdowns. Cells were infected (MOI=0.001) 48 h post-siRNA transfection for 20 h, stained with CellTracker Orange, and imaged to determine the fold-change in GFP signal compared to Luc siRNA (control) treatments. C. Fold-change in GFP reporter readout following VSVM51R-GFP infection of U2OS cells after indicated knockdowns. Cells were infected (MOI=0.0001) 48 h post-siRNA transfection for 20 h, stained with CellTracker Orange, and imaged to determine the fold-change in GFP signal compared to Luc siRNA (control) treatments. D. Cell counts of control or U2OSΔRNF214 cells over a 72 h time course. E-F. Results of cytotoxicity assays in control or U2OSΔRNF214 cells infected at increasing MOIs of VSVM51R-GFP for 20 h. Following infection, supernatants were collected for LDH-based cytotoxicity assays (E) and cells were lysed for CellTiter-Glo-based cell viability assays (F). “Lysed” refers to cells lysed in lysis buffer to serve as a positive control for cell death. G-I. Fold-change in IFNB1 (G), IFIT1 (H), and RSAD2 (Viperin) (I) transcript levels (compared to mock-infected control cell levels) in control or U2OSΔRNF214 cells infected with VSVM51R-GFP (MOI=0.0001) for 20 h. IFNB1 encodes IFN-β whereas IFIT1 and RSAD2 are IFN-stimulated genes [39, 40]. J. Representative immunoblot of endogenous human IFIT3 and ISG15 (which are encoded by IFN-stimulated genes [40]) levels following treatment of control or U2OSΔRNF214 cells with increasing doses of recombinant IFN-β. Data in B-I are means ± SEM; n=3. Statistical significance for B,C and the multiple comparisons of G-I was determined with One-way ANOVA and Dunnett’s post-test; ns (not s [file ppat.1013035.s006.tif]

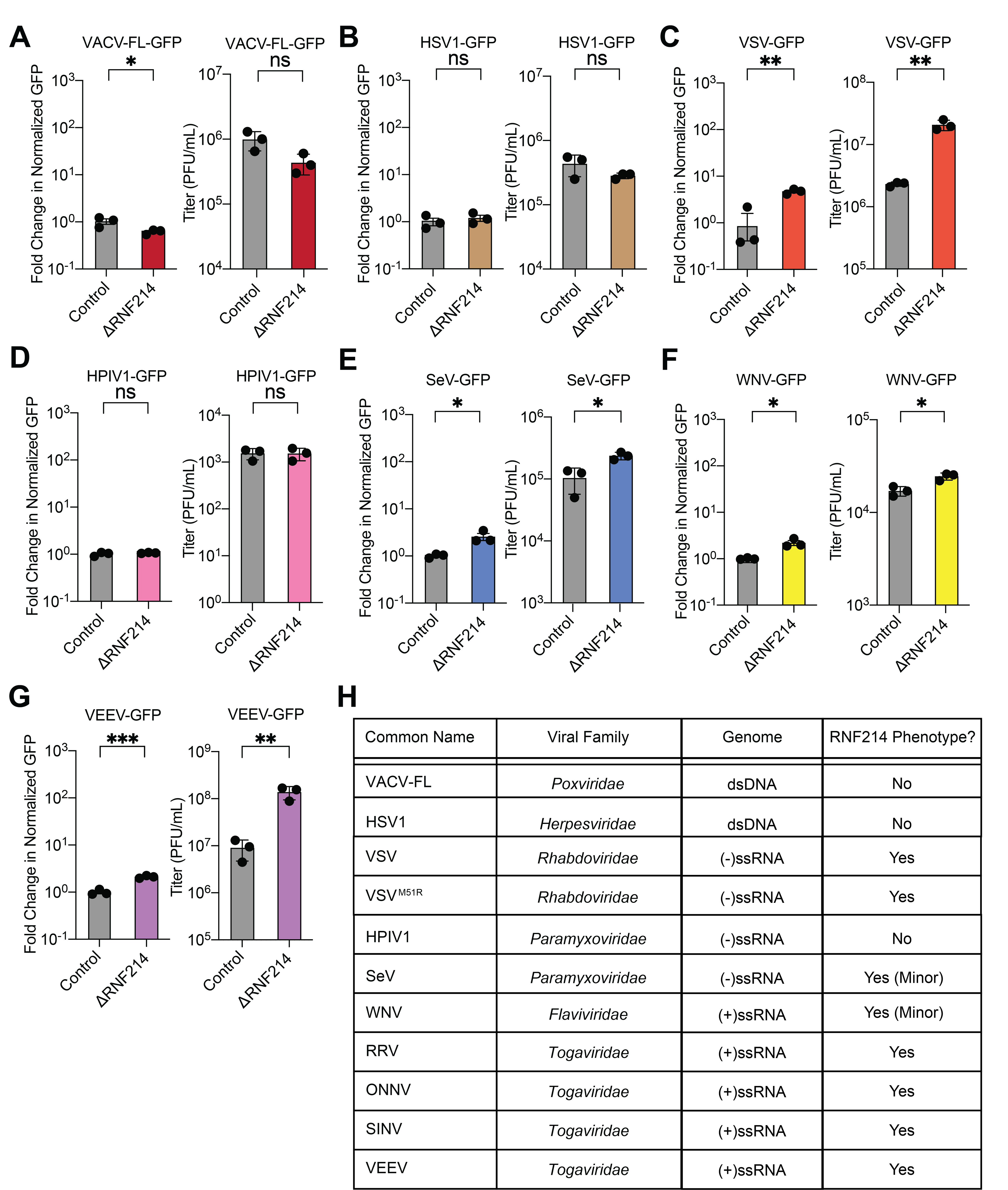

Supplement: S4 Fig — Assessing the impact of RNF214 knockout on the replication of diverse viral families. A-B. Control or U2OSΔRNF214 cells infected with the indicated reporter poxvirus (VACV-FL-GFP; MOI=0.01) or herpesvirus (HSV-1-GFP; MOI=0.01). Following infection, cells were stained with CellTracker Orange and imaged for fold-change in GFP reporter readout compared to control cells. Titers are also shown from these collected cultures. C-G. Similar experiments were performed as in A-B but with VSV-GFP (MOI=0.0001) (C), the paramyxoviruses: HPIV1-GFP (MOI=0.001) (D) and SeV-GFP (MOI=0.001) (E), the flavivirus, WNV-GFP (MOI=0.001) (F) and the togavirus, VEEV-GFP (MOI=0.001) (G). H. Table summarizing results for the various viral families tested in this figure. Data for A-G are means ± SEM; n=3. Statistical significance was determined with unpaired Student’s t-test; ns (not significant), *=P<0.05, **=P<0.01, ***=P<0.001, ****=P<0.0001. (TIF) [file ppat.1013035.s007.tif]
